# Influenza and other respiratory viral infections associated with absence from school among schoolchildren in Pittsburgh, Pennsylvania, USA: a cohort study

**DOI:** 10.1186/s12879-021-05922-1

**Published:** 2021-03-22

**Authors:** Jonathan M. Read, Shanta Zimmer, Charles Vukotich, Mary Lou Schweizer, David Galloway, Carrie Lingle, Gaby Yearwood, Patti Calderone, Eva Noble, Talia Quadelacy, Kyra Grantz, Charles Rinaldo, Hongjiang Gao, Jeanette Rainey, Amra Uzicanin, Derek A. T. Cummings

**Affiliations:** 1 Center for Health Informatics Computing and Statistics, Lancaster Medical School, Lancaster University, Lancaster, UK; 2 Institute of Infection and Global Health, University of Liverpool, Liverpool, UK; 3 Division of General Internal Medicine, University of Pittsburgh School of Medicine, Pittsburgh, PA USA; 4 Department of Medicine, University of Colorado School of Medicine, Denver, CO USA; 5 University of Pittsburgh Graduate School of Public Health, Pittsburgh, PA USA; 6 Toledo Lucas County Health Department, Toledo, OH USA; 7 Department of Anthropology, University of Pittsburgh, Pittsburgh, PA USA; 8 Department of Epidemiology, Johns Hopkins Bloomberg School of Public Health, Baltimore, MD USA; 9 Department of Biology, University of Florida, Gainesville, FL USA; 10 University of Florida, Emerging Pathogens Institute, Gainesville, FL USA; 11 Department of Global Migration and Quarantine, US Centers for Disease Control and Prevention, Atlanta, GA USA

**Keywords:** Influenza, human, Schools, Child, Prevention & Control, Epidemiology

## Abstract

**Background:**

Information on the etiology and age-specific burden of respiratory viral infections among school-aged children remains limited. Though school aged children are often recognized as driving the transmission of influenza as well as other respiratory viruses, little detailed information is available on the distribution of respiratory infections among children of different ages within this group. Factors other than age including gender and time spent in school may also be important in determining risk of infection but have been little studied in this age group.

**Methods:**

We conducted a cohort study to determine the etiology of influenza like illness (ILI) among 2519 K–12 students during the 2012–13 influenza season. We obtained nasal swabs from students with ILI-related absences. Generalized linear mixed-effect regressions determined associations of outcomes, including ILI and laboratory-confirmed respiratory virus infection, with school grade and other covariates.

**Results:**

Overall, 459 swabs were obtained from 552 ILI–related absences. Respiratory viruses were found in 292 (63.6%) samples. Influenza was found in 189 (41.2%) samples. With influenza B found in 134 (70.9%). Rates of influenza B were significantly higher in grades 1 (10.1, 95% CI 6.8–14.4%), 2 (9.7, 6.6–13.6%), 3 (9.3, 6.3–13.2%), and 4 (9.9, 6.8–13.8%) than in kindergarteners (3.2, 1.5–6.0%). After accounting for grade, sex and self-reported vaccination status, influenza B infection risk was lower among kindergarteners in half-day programs compared to kindergarteners in full-day programs (OR = 0.19; 95% CI 0.08–0.45).

**Conclusions:**

ILI and influenza infection is concentrated in younger schoolchildren. Reduced infection by respiratory viruses is associated with a truncated school day for kindergarteners but this finding requires further investigation in other grades and populations.

**Supplementary Information:**

The online version contains supplementary material available at 10.1186/s12879-021-05922-1.

## Background

The 2009 influenza pandemic caused disruption to schools, businesses, and governmental entities. Numerous reports from US CDC and European countries document the central role of school-aged children in community-wide transmission of pandemic virus [1–10]. In the United States and Australia, the onset of pandemic influenza incidence was linked to school opening dates [11]. Children experience higher rates of infection, shed influenza virus longer than adults, and have social mixing patterns conducive to the propagation of respiratory viruses [5, 12]. However, the risk distribution of influenza and other respiratory infections throughout childhood is poorly understood. Current influenza vaccination programs in the United States target individuals 6 months of age and older. If vaccine supply is limited, children 6–59 months of age are an age group of focus for vaccination [13]. Programs in the United Kingdom target children 17 years of age and younger, with a program that phases universal coverage in beginning with younger children [14].

A number of seasonal pathogens cause respiratory symptoms consistent with influenza-like illness (ILI) [15]. Given the importance of school-aged children for influenza transmission, a better understanding of the etiology of respiratory infections among children, their associated burden, and the interaction of these infections with influenza is required to improve current public health strategies to reduce transmission of infections in schools and communities.

Despite the role of school-aged children in driving influenza epidemics, relatively little fine-grained analysis has been conducted to assess how different ages within the school demographic are affected by seasonal influenza [16]. Factors other than age may also be important in determining risk of infection. Females have been shown to be at greater risk of influenza infection in adults [17], but it is unknown if school-aged females are also at elevated risk compared to male pupils in the same schools. Small-scale spatial variation in influenza attack rate has been observed [18], though it is unclear whether nearby schools (within the same district) experience similar epidemic dynamics. The acquisition of immunity during childhood is thought to drive patterns of age specific incidence of influenza [19]; few detailed studies have examined how infection risk changes with age during childhood.

We conducted the SMART study (Social Mixing And Respiratory Transmission study) in two school districts in the Pittsburgh metropolitan area. This study included testing school-aged children for respiratory viruses following an ILI-related absence during the winter influenza season of 2012–2013 in a cohort of schools. We relate infection outcomes to attributes of individual children and grade and classroom properties, as well as school districts and the individual schools within them.

## Methods

### Study population

We conducted our study in a charter school system in an urban area (district A: eight schools and 2000 kindergarten to 12th grade [K-12] students) and a public school district in a suburban area surrounding an urban core (district B: nine schools and approximately 4700 K-12 students). We conducted a purposive sample of schools to study a selection that broadly represented the diversity of socioeconomic and racial compositions of public schools across the Pittsburgh metropolitan area. School districts and schools were approached and those willing to participate were selected for inclusion. We approached only two school districts, and both agreed to participate. We worked with nine schools selected from these two districts: three from district A and six from district B.

### Recruitment and study procedures

Project staff met with schoolboards, parent-teacher organizations, school staff, and school nurses to provide information about the project. Investigators provided students and parents a summary of the study, including a disclosure form and a signature section for opting out of participation. All parents received information sheets ahead of the study period, offering them the option to withdraw their child from the study. All participating children gave verbal assent prior to swabbing and interviewing.

### Surveillance and virologic sampling

The 2012–13 influenza transmission season started unusually early nationwide [20]. In schools participating in our study, influenza cases began to rise in mid-December. The SMART study team initiated surveillance in three schools during the week of December 17–21, 2012, prior to the scheduled winter break, and in the first week of January 2013. Surveillance activities were implemented in all nine participating schools from the start of the spring semester on January 7, 2013, concluding on March 27, 2013. Schools closed for winter break December 24, 2012 to January 2, 2013; spring break closure was March 28 to April 1, 2013.

Trained project staff were deployed in participating schools to monitor student absentee data and receive daily attendance reports from schools. We defined an absence event as absence from school by a student for either an entire day or missing part of the day due to leaving school early due to illness. This excluded individuals who were tardy but present for part of the day. Students with influenza-like illness at school either self-identified or were identified as ill by their teachers and sent to a school nurse. School nurses were present in all schools. School nurses contacted the parents of ill children to coordinate treatment as well as to confirm parental assent for swabbing of their children. Children of assenting parents were swabbed for virological testing either on presentation to the school nurse when ill or on return to school when ILI was identified through contact of parents. All contiguous days for which a student was absent were classified as a single absence event. Study staff telephoned parents of absent children to determine the reason for student absences. If an absent student was reported ill, staff then inquired about the symptoms the student had (categories recorded were: fever, sore throat, cough, runny nose/congestion, headache, muscle or joint pain, nausea/diarrhea/vomiting). Students with ILI using the CDC Case Definition - fever of at least 37.8 °C, and either cough or sore throat - were eligible for nasal swabbing by project or school staff upon return to school. Temperature was requested and recorded for those individuals who reported a recorded temperature, however, to increase sensitivity of case finding, children that a parent or guardian reported as having a fever, even in the absence of a measurement were included as meeting our case definition. Students presenting to the school nurse with ILI were swabbed immediately, otherwise students were swabbed on their return to school following illness absence.

### Sampling and laboratory analysis

Staff used sterile wound polyester-tip swabs to collect specimens from the anterior nares of participants; the swab was placed in sterile transport media, and transported to University of Pittsburgh Medical Center Clinical Virology Laboratory, where PCR tests were performed for; influenza A/H1 and influenza A/H3 (Influenza A), influenza B (Influenza B), RSV A and B (RSV), rhinovirus (HRV), coronavirus 229E, NL63, HKU1 and OC43 (CV), and adenovirus B, C and E (ADNO) (see supplement for further details).

### Questionnaire

Participating students were surveyed on a repeated basis to determine influenza vaccine status.

### Statistical analysis

Cumulative attack rates (CARs) were calculated from pooled data across all schools and the entirety of the surveillance period. CARs were calculated for participants who were identified as: 1) having ILI, 2) testing positive for any of the respiratory viruses in the panel, or 3) testing positive for influenza virus, regardless of subtype and testing positive for each of subtypes of influenza separately. Denominator values included the number of participating students, students with absence periods who reported to the school nurse, and students with absence periods who did not report to the school nurse but whose home we were able to contact. We excluded students whose homes we were not able to contact to determine their health status even after multiple attempts. Binomial confidence intervals were calculated for all CARs.

We performed a variety of generalized linear mixed-effect regressions, in which outcome variables were the binary infection status of a participant over the entire surveillance period given a particular definition of outcome: whether they were identified as having ILI; testing positive for any respiratory virus; testing positive for influenza; or testing positive for a specific subtype of influenza (A and B). We included hierarchical random intercept terms for school and district in all models unless specified otherwise, where schools were nested within one of the two districts. We performed hypothesis-driven regression models, in which the variables included in the model were decided a priori. These included: a linear term for a child’s school grade (to explore the effect of increasing age, where Kindergarten grade is replaced with a zero and grade treated as a numeric term), attendance duration (full-day or half-day, to explore the effect of reduced time spent at school), sex (to identify any sex difference in risk within this age group), and vaccination status (to estimate vaccine efficacy). Random effect terms for school and district were included to identify variation in risk between schools and districts. To assess the robustness of our hypothesis-driven modelling, we also performed an exploration of alternative models using a forward selection process in which the final model’s variables were selecting from a series of candidate variables including those listed above as well as a the percentage of students eligible for free or reduced price lunch (school level), social mixing rate and class size (student level) (described in supplement; Tables S1, S2, S3 and S4).

## Results

### Recruitment

A total of 2519 students participated in the study at one elementary school (grades K-6), a combined school (grades K-8), and a high school (grades 9–12) from the charter district–District A, and from five elementary schools (grades K-4) and an intermediate school (grades 5–6) in the public school district–District B (Table 1 and S0). As there were only 12 students in the 12th grade, we combined grades 11 and 12 for all analyses (termed ‘11+’). We achieved good participation rates across the schools: 95.2% in school A1, 99.6% in school A2; 96.9% in school A3; 76.5% in school B1; 89.6% in school B2; 85.0% in school B3; 90.0% in school B4; 84.5% in school B5; 86.8% in school B6. An additional three students dropped out during the study period. After accounting for children for whom we could not achieve follow-up following an absence (see Absenteeism section below), we were able to successfully surveil 2077 children during the study period.

**Table 1:** Demography of the study population

	Total	Grade											
		K	1	2	3	4	5	6	7	8	9	10	11+
Total study population	2519	314	319	343	355	351	227	275	46	38	88	90	79
Observable for ILI (%)													
Yes	2077 (82)	279 (88)	267 (84)	300 (87)	301 (85)	304 (87)	185 (81)	216 (79)	34 (74)	30 (79)	61 (69)	50 (55)	50 (63)
No	442 (18)	35 (22)	46 (26)	43 (23)	54 (15)	47 (23)	42 (19)	59 (21)	12 (26)	8 (21)	27 (31)	40 (45)	29 (37)
Observable population													
District													
A	647	54	53	62	56	66	62	69	34	30	61	50	50
B	1430	225	214	238	245	238	123	147	–	–	–	–	–
School													
A1	204	27	26	32	21	32	30	36	–	–	–	–	–
A2	161	–	–	–	–	–	–	–	–	–	61	50	50
A3	282	27	27	30	35	34	32	33	34	30	–	–	–
B1	201	40	40	36	50	35	–	–	–	–	–	–	–
B2	270	–	–	–	–	–	123	147	–	–	–	–	–
B3	240	47	43	54	41	55	–	–	–	–	–	–	–
B4	188	36	34	44	33	41	–	–	–	–	–	–	–
B5	342	67	60	59	85	71	–	–	–	–	–	–	–
B6	189	35	37	45	36	36	–	–	–	–	–	–	–
Sex													
Female	995	145	125	149	137	136	91	97	14	17	34	27	23
Male	1082	134	142	151	164	168	94	119	20	13	27	23	27
Self-reported vaccination §													
No	850	133	111	131	118	122	74	63	17	10	22	25	24
Yes	694	63	95	105	107	120	66	74	10	8	20	13	13
Not reported	533	83	61	64	76	62	45	79	7	12	19	12	13

**§** Of 2077 participants, we obtained a valid immunization response from 1544 children: we failed to interview 296 children regarding their vaccination status; of the children interviewed, 237 did not know their vaccination status or refused to provide a valid answer for any of the times they were questioned

### Absenteeism

In total, 1772 children generated 4720 absence events during the study period. We were unable to successfully call the home and determine the health status of 442 (24.9%) absent children who did not present to the school nurse with illness; collectively, they were responsible for 970 (20.6%) absence events. Our ability to successfully characterize illness in children following absence was greatest for schools in district A and among younger children (Table S2), We were able to characterize fully 3750 absence events absences among 1330 children. Older children (grades 7–12) had fewer absences with ascertained cause (i.e. observable for ILI outcome) (see Table 1).

### Time course of ILI and confirmed infections

**Fig. 1:**
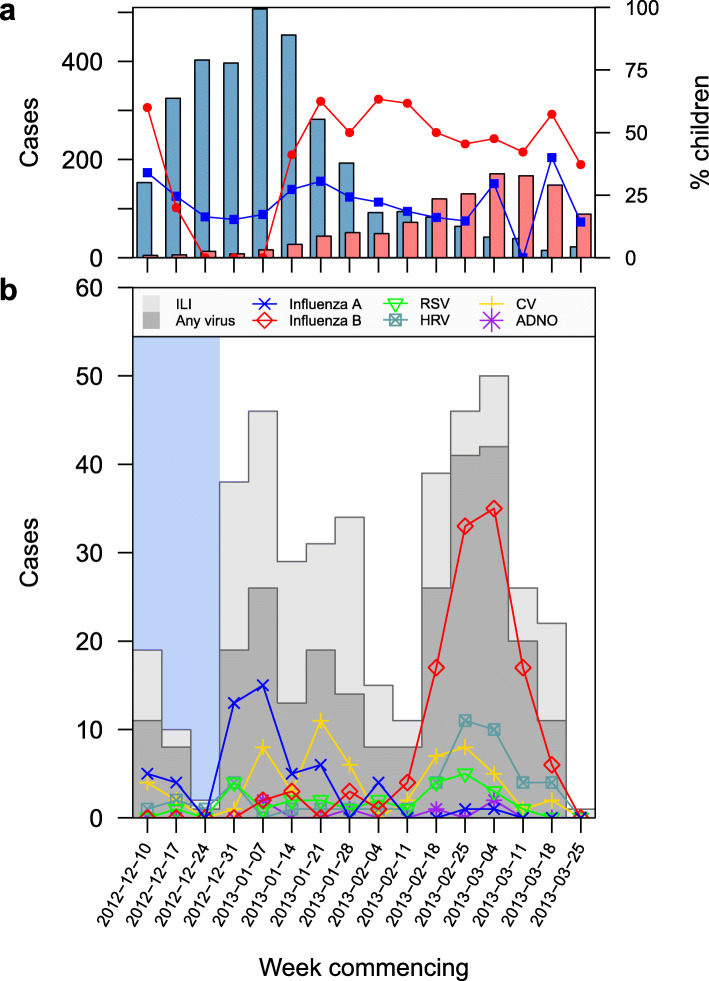
Time series of cases during 2012–2013. **a** Weekly influenza cases reported during the study period by Pennsylvania Health Department for Allegheny county (emergency department and outpatient provider-reported), stratified by influenza type (blue bars, influenza A; red bars, influenza B). Also shown are the percentage of cases who are children (< 16 years old); blue and red lines represent influenza A and B respectively. **b** Cases of illness and infection recorded by the study. The pale blue region indicates the period (during 2012) in which only three schools were participating in the study. Numbers of students matching the definition of ILI are denoted by light grey bars; those with virologically confirmed virus are denoted by dark grey bars. Numbers of students testing positive for specific respiratory viruses are shown by the lines and are grouped as follows: influenza A/H1 and influenza A/H3 (Influenza A); influenza B (Influenza B); RSV A and B (RSV); rhinovirus (HRV); coronavirus 229E, NL63, HKU1 and OC43 (CV); adenovirus B, C and E (ADNO)

Of the children who were absent from school or reported to the school nurses, 408 were diagnosed with ILI and swabbed, of which 271 children were PCR positive for at least one virus. Some children had multiple ILI episodes and were swabbed more than once: 39 were swabbed twice, and six swabbed three times. Of those 271 children testing positive at least once with confirmed virus, 180 (66.4%) were positive for influenza (either subtype A or B), while 56 (20.7%) and 132 (48.7%) were positive for influenza A and B, respectively, during the surveillance period. A small wave of influenza A preceded a larger wave of influenza B cases, corresponding to distinct epidemics of A and B occurring in the wider community (Fig. 1). A substantial proportion of children with were positive for respiratory pathogens other than influenza. Among the children testing positive for any virus, 64 (23.6%) were positive for coronavirus, 54 (19.9%) rhinovirus, 28 (10.3%) respiratory syncytial virus (RSV), 6 (2.2%) adenovirus, 3 (1.1%) picornavirus, and 3 (1.1%) metapneumovirus.

### Cumulative attack rates (CARs)

**Fig. 2:**
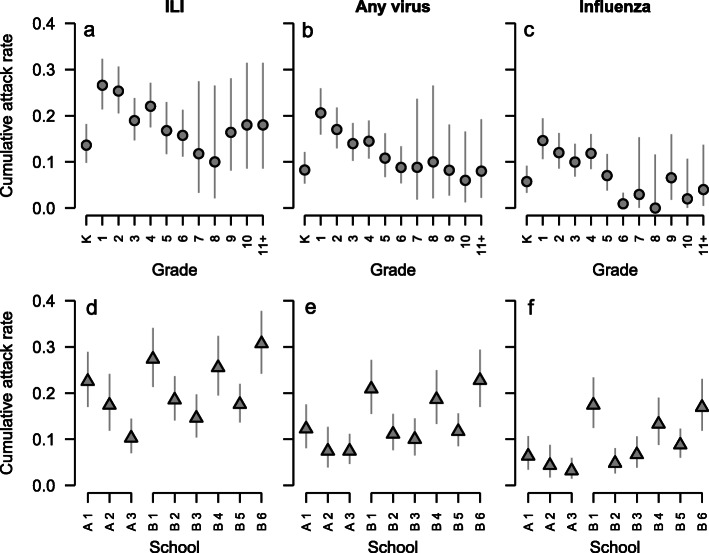
Cumulative attack rate, by grade and school, respectively, for all children diagnosed with influenza-like illness symptoms (ILI) **a**, **d**, those that are laboratory-confirmed with any of the respiratory viruses tested **b**, **e**, and those positive for influenza virus **c, f**. Lines denote binomial 95% confidence intervals. Schools are arranged by district (A1 to A3, B1 to B6)

In our study, we found an overall cumulative attack rate of 19.7% (95% CI, 18.0–21.5%) for ILI, 13.1% (11.7–14.6%) for infection with a respiratory virus, and 8.7% (7.5–10.0%) for influenza infection. We found significant variation in CAR between grades and schools for children identified with ILI, children testing positive for any of the viruses tested, and children testing positive for influenza (Fig. 2; Figs. S1, S2, S3). The highest rate of ILI was in grades 1 (26.6, 95% CI 21.4–32.3%) and 2 (25.3, 20.5–30.7%), while the rate ranged between 10.3% (95% CI, 7.0–14.4%) and 30.7% (24.2–37.8%) between schools. Children in grade 1 had the highest CAR for all viruses (20.6, 95% CI 15.9–26.0%), and the rate decreased with increasing grade, although kindergarten children had a lower CAR than children in grade 1 (8.2, 95% CI, 5.3–12.1%). We also found significant differences in CAR for infection by any respiratory virus between the schools (Fig. 2b). For influenza, similar patterns were observed: there were significant differences in CAR between grades and between schools, with the highest CAR being in younger aged children in grades 1 to 4, and CAR ranging in individual schools from 3.2% (95% CI, 1.5–6.0%) to 17.4% (95% CI, 12.4–23.4%). There were different patterns of CAR for influenza A and B (Fig. S1) and other respiratory viruses across grades and schools (Fig. S2), and across grade within the same school (Fig. S3).

### Sampling delays

Long delays between symptom onset and sampling may introduce bias in the surveillance of acute respiratory viruses, as viral shedding rates diminish as individuals recover. While the delay between symptom onset and swabbing in our study ranged between 0 and 22 days, the median delay was 4 days and 83% of samples were taken within 6 days of symptom onset. Overall, we found no strong indication that positivity rates declined with increased delay in sampling (Fig. S4, Table S8). We also found no secular trends in sampling delays (Fig. S5).

### Regression models

CARs are a crude measure of true infection rates, due to the potential for confounders and the pooling of data across all participating schools. To explore the impact of multiple factors in influencing the infection rates of children, we fitted a series of mixed-effect regression models that explicitly incorporated the hierarchical nature of the observations within schools and districts. We tested whether case status (either having ILI, any virus, influenza or specific influenza subtype) was associated with the sex, school grade, self-reported vaccination status of students, or the duration of their instruction at school (full day vs half day). Half day duration only occurred in one of the school districts studies (district B).

We found broad agreement in the effect sizes of covariates and random variables across the five modelled outcomes (Table 2). Across all the different models, there was greater variation between schools than between districts. The proportion of variation associated with schools ranged from 11.2% (respiratory virus infection) to 26.5% (influenza B infection). Increasing school grade, a proxy for age, was associated with a reduction in risk in all outcomes. We did not find a significant effect of sex in any of the models. Vaccination status was not associated with viral infection risk but was associated with a reduction in the risk of ILI. Kindergarten children attending school for a half day were at significantly reduced risk of all infection outcomes except for influenza A infection. Half day attendance was most strongly associated with the risk of influenzas B infection, representing a reduction in risk of between 55 and 92%. These associations were supported further when we used a model selection process to consider alternative model formulations (Tables S3 and S4; supplementary information). An additional variable, percentage of students eligible for free or reduced price lunch, was selected in final models for all outcomes except influenza A. The association in all these models suggests students in schools with higher percentages of students eligible for free or reduced price lunch tend to be at reduced risk of ILI, respiratory virus and influenza B infections.

**Table 2:** Odds ratios^$^ of ILI, any virus, influenza (A or B), influenza A, influenza B on length of 445 school instruction, grade, sex, and 446 self-reported vaccination status among schoolchildren in Pittsburgh, PA. Variables significant at the 95% level are shown in bold

	Response variable^*^				
	Symptomatic infection	Laboratory confirmed infection			
	ILI	Any respiratory virus^**%**^	Influenza (A or B)	Influenza A	Influenza B
**Random effect variables**	Percent of variance attributable to random effect^				
School	12.8	11.2	20.1	17.0	26.5
District	0.0	1.5	1.9	0.0	9.6
Residuals	87.2	87.3	78.0	83.0	63.9
**Fixed effect variables**	Odds ratio (95% Confidence Intervals)				
Intercept	**0.43 (0.29–0.65)**	**0.28 (0.16–0.49)**	**0.19 (0.09–0.40)**	**0.06 (0.03–0.14)**	**0.10 (0.04–0.25)**
Duration of attendance					
Full day (*n* = 1880)	1	1	1	1	1
Half day (*n* = 197)	**0.35 (0.21–0.57)**	**0.28 (0.15–0.51)**	**0.20 (0.10–0.43)**	0.38 (0.12–1.17)	**0.19 (0.08–0.45)**
Grade (linear term)	**0.93 (0.87–1.00)**	**0.91 (0.84–0.99)**	**0.85 (0.77–0.95)**	**0.85 (0.72–0.99)**	**0.88 (0.78–1.00)**
Sex					
Male (*n* = 1082)	1	1	1	1	1
Female (*n* = 995)	0.91 (0.73–1.13)	0.81 (0.62–1.05)	0.94 (0.69–1.29)	1.00 (0.59–1.70)	0.90 (0.63–1.29)
Vaccination					
No (*n* = 850)	1	1	1	1	1
Yes (*n* = 694)	**0.70 (0.54–0.90)**	0.80 (0.59–1.08)	0.81 (0.57–1.17)	0.59 (0.32–1.12)	0.90 (0.60–1.36)
Not reported (*n* = 533)	0.76 (0.58–1.01)	0.74 (0.53–1.03)	0.80 (0.54–1.20)	0.63 (0.32–1.25)	0.86 (0.54–1.37)

$ Odd ratios were calculated using random effect logistic regression

^%^ Influenza A/H1, Influenza A/H3, Influenza B, RSV A, B, Picornavirus 1, 2, 3, 4, Metapneumovirus, Rhinovirus detected, Adenovirus B, C, E, Coronavirus 229E, NL63, HKU1, OC43 detected using Genmark Diagnostic’s RVP-RUO panel

^ Percentage of variance attributable to each random effect was calculated by dividing the standard deviation of each component by the total

### Additional analyses of half-day kindergarten attendance

To further explore the impact of half-day school attendance, we fitted models which explore the association between grade (Table S5) and duration of attendance (Table S6) and infection outcome in schools with half day kindergarten attendance. We also fitted models of infection outcomes with variables for attendance duration, grade, sex and vaccination status, in schools which have both full day and half day kindergarten students (Table S7). We found Kindergarten students at significantly reduced risk of infection compared to higher (older) grade students in schools with half day attendance (Table S5). However, in the same schools, an analysis that did not include grade and looked only at half day attendance, we did not find a statistically significant reduction in risk associated with half day attendance (Table S6). Thus, our results indicate that accounting for decreasing risk with increasing grade was important to detecting a statistically significant effect associated with half day status. When we fitted the full model (including terms for attendance, grade, sex and vaccination status) to the four schools in our study with both half and full day kindergarten students, we found half day kindergarten students were at significantly reduced risk of all infection outcomes (Table S7), though we did not find a significant association between infection risk and grade in these models.

## Discussion

We conducted surveillance for influenza-like illness within nine schools during a single influenza season (2012–2013). Nearly 20% of surveilled children experienced at least one episode of ILI during the study period; several experienced multiple episodes. Influenza was the most common viral pathogen identified among these children, although we found the presence of other respiratory viruses in a substantial proportion of detected ILI episodes, suggesting that the burden of ILI in school age children during the normal influenza season may not be entirely due to infection with influenza. ILI and infections were concentrated in younger children, and CARs varied between schools. Modelling of the risk of infection with respiratory viruses and, specifically, influenza identified associations with the grade and the attendance duration of children, where younger children are at higher risk of infection than older children, and kindergarten children attending school for half days were at lower risk than kindergarten children attending for full day instruction.

Contacting parents of absent students to document reasons for absence was a major challenge during the study. However, our rate of follow-up for an absence episode (47.2% of cases) compared favorably to an earlier study in which only 28.6% of absences were verified [21], though we did observe variation in documentation rates by school and grade. The primary problem in contacting parents was inaccurate phone numbers. This may be associated with the socio-economic status of the parents (and likely the school catchment). Nonetheless, the pattern of influenza-associated absences confirmed during the study period mirror emergency department and outpatient provider-reported, virologically confirmed influenza from the Allegheny County Department of Health, suggesting that absentee-triggered surveillance was an effective methodology (Fig. 1).

Our study found overall CARs for respiratory virus infection of 13.1% (95% CI, 11.7–14.6%) and for influenza infection of 8.7% (95% CI, 7.5–10.0%); these rates are consistent with those found in other studies of children in non-clinical settings [16]. Our study also shows that the cumulative attack rate of respiratory viruses decreases with increasing grade of school-aged children. This age pattern of infection suggests that respiratory infectious disease prevention measures, including efforts to further increase seasonal influenza vaccine uptake, should be especially emphasized for the younger age groups: kindergarten through grade 6. We found the strongest association of grade and time spent in school on the risk of infection with influenza B; this was the outcome with the largest number of cases and, thus, the greatest power to detect significant associations. We found infection outcomes were clustered within schools but varied little between districts, after adjusting for other factors. This observation is consistent with a greater risk of influenza due to school-based rather than community-based transmission in our study population, though the clustering of other risk factors in schools could also explain our results. In the model selection process, an additional variable (percentage of students within a school eligible for free or reduced price lunch) was selected for several of the infection outcomes: lower percentages were associated with increased risk of ILI, respiratory virus and influenza B. This may reflect higher levels of immunity generated by greater exposure to viral pathogens earlier in life, or reduced detection of viral infections in our study through reduced access of study staff to parents to obtain reasons for absence or consent to participate. Our hypothesis driven models accounted for the potential for differences in infection rates between schools through random effects. The consistency of our estimated coefficients in hypothesis driven models and ‘agnostic’ selected models increases our confidence that school and district level random effects accounted for systematic differences between schools that may be driven by socio-economic factors or other unmeasured demographic variables.

Kindergarten students who attended school for half-day duration were at significantly lower risk of respiratory infection suggests the reduced risk of infection in kindergarten students may be due to reduced time spent within school. Further study is required to determine if these findings can be replicated in other settings, and if half-day school attendance would similarly affect respiratory viral infection in other grades. It is possible that our results reflect the experiences and exposures of kindergarten students within a single district and may not be generalizable to other kindergarten students or other age groups and school grades. Nonetheless, the prospect of a truncated school day as a potential alternative to full school closure, as a control option to reduce the transmission of seasonal and pandemic influenza [4, 22], is intriguing. Pre-emptive, school closures are recommended as a countermeasure during severe influenza pandemics [23], but are associated with significant educational, social and economic impacts [24]. Prior studies of the impact of school closures on respiratory viral transmission have focused on observations around planned and unplanned closures [4, 6, 25], but none have characterized grade specific differences or half-day attendance. Further work is required to assess the efficacy of a half-day attendance regime in non-kindergarten grades and in other school populations on influenza infection and transmission.

There are several important limitations to this study. Our detection of virus relied on identification of an ILI episode within a child; we may, therefore, have underestimated the of incidence of viral infections, particularly if infections can be asymptomatic. While we found little evidence of an effect of vaccination status on infection status, we relied on the children to self-report their vaccination status. This may have introduced bias through misclassification error and masked any true effect vaccines may have had in altering individual infection risk. Our findings may also be biased due to differences in our ability to characterize illness in absent children from different ages and schools. Follow-up for absence was harder to achieve in schools with higher percentage students eligible for free or reduced-price lunches, indicating a possible socio-economic reason. ILI and infection in older children and in schools with students from poorer backgrounds may, therefore, be underestimated in our study. A further limitation is that we have not measured other variables that confound or better explain the observation that half-day kindergarten children are at reduced risk of infection. Half-day kindergarten enrollment may be associated with socioeconomic status of families that may in turn be associated with the risk of acquisition and/or detection of respiratory viral illness in our study. The availability of half-day kindergarten programs by school may also be associated with socioeconomic characteristics of our study locales that could be associated with our outcomes. Kindergarten children in particular schools were reported by the school as attending for either a half or full school day; we had no further information on the time actually spent in school or daycare by these individuals.

## Conclusions

Our study found ILI and respiratory infections to be common among school age children during the 2012–2013 influenza season, with the youngest children being at highest risk of infection with influenza. We found no evidence of a difference in infection risk between sexes. We found a shortened school day to be associated with a reduced risk of infection, but this effect was only observed in kindergarteners.

## Supplementary Information

**Additional file 1: Table S0**. Demographic characteristics of study schools. We present the number of participating and observed students. Distinction is made between kindergarten students enrolled in full-time and half-day programs in schools which operate such programs. **Table S1**. Grade-specific contact rates. **Table S2**. Unobservable students with (enrolled students for whom we could not complete a telephone follow-up upon their absence) and number of absence and characterizable absence events made by enrolled students, stratified by school, grade and sex. **Table S3**. Forward model selection process. **Table S4**. Final models identified using the forward selection process. **Table S5**. Odds ratios$ of ILI, any virus, influenza (A or B), influenza A, influenza B on grade within all schools where kindergarteners were taught for half-days (schools B1, B3, B4, B5, and B6). **Table S6**. Odds ratios$ of ILI, any virus, influenza (A or B), influenza A, influenza B on duration of instruction within schools with any kindergarteners taught for half-days (schools B1, B3, B4, B5, and B6). **Table S7**. Odds ratios$ of ILI, any virus, influenza (A or B), influenza A, influenza B on length of school instruction, grade, sex, and self-reported vaccination status among schoolchildren in schools with both half and full day Kindergarteners (schools B1, B3, B4, and B5). As all the schools belonged to the same school district, only a random effect term for school was included in the models. Variables significant at the 95% level are shown in bold. **Table S8**. Association between testing positive according to four different outcomes and delay in swabbing (delay between symptom onset and swab taken). **Figure S1**. Cumulative attack rate for influenza type A and B, by grade and district. **Figure S2**. Cumulative attack rates, stratified by grade and school, for other respiratory viruses. **Figure S3**. Cumulative attack rate (CAR) by infection outcome (columns), stratified by grade and school (rows). **Figure S4**. Relationship between the proportion of samples testing positive for virus and the delay between symptom onset date and swabbing date. **Figure S5** Spline of secular time fitted using a negative binomial GAM of swabbing delay.

## Data Availability

Raw data used in this analysis are available, upon reasonable request, from the corresponding authors.

## Abbreviations

ADNO: Adenovirus
°C: Degrees Celsius
CARs: Cumulative attack rates
CV: Coronavirus
HRV: Human rhinovirus
ILI: Influenza like illness
IRB: Institutional review board
K: Kindergarten
OR: Odds Ratio
PCR: Polymerase chain reaction
RSV: Respiratory syncytial virus
SMART study: Social Mixing And Respiratory Transmission study
US CDC: United States Centers for Disease Control and Prevention
