# Supplementary material for: Influenza and other respiratory viral infections associated with absence from school among schoolchildren in Pittsburgh, Pennsylvania, USA: a cohort study
Source: BMC Infect Dis. 2021 Mar 22;21:291. doi: 10.1186/s12879-021-05922-1 (PMC7983083; doi:10.1186/s12879-021-05922-1)
Supplement: Supplementary file 1 — Additional file 1: Table S0. Demographic characteristics of study schools. We present the number of participating and observed students. Distinction is made between kindergarten students enrolled in full-time and half-day programs in schools which operate such programs. Table S1. Grade-specific contact rates. Table S2. Unobservable students with (enrolled students for whom we could not complete a telephone follow-up upon their absence) and number of absence and characterizable absence events made by enrolled students, stratified by school, grade and sex. Table S3. Forward model selection process. Table S4. Final models identified using the forward selection process. Table S5. Odds ratios$ of ILI, any virus, influenza (A or B), influenza A, influenza B on grade within all schools where kindergarteners were taught for half-days (schools B1, B3, B4, B5, and B6). Table S6. Odds ratios$ of ILI, any virus, influenza (A or B), influenza A, influenza B on duration of instruction within schools with any kindergarteners taught for half-days (schools B1, B3, B4, B5, and B6). Table S7. Odds ratios$ of ILI, any virus, influenza (A or B), influenza A, influenza B on length of school instruction, grade, sex, and self-reported vaccination status among schoolchildren in schools with both half and full day Kindergarteners (schools B1, B3, B4, and B5). As all the schools belonged to the same school district, only a random effect term for school was included in the models. Variables significant at the 95% level are shown in bold. Table S8. Association between testing positive according to four different outcomes and delay in swabbing (delay between symptom onset and swab taken). Figure S1. Cumulative attack rate for influenza type A and B, by grade and district. Figure S2. Cumulative attack rates, stratified by grade and school, for other respiratory viruses. Figure S3. Cumulative attack rate (CAR) by infection outcome (columns), stratified by grade and school (rows). Figure S4. Relati [file 12879_2021_5922_MOESM1_ESM.docx]

**Supplementary material**

**Laboratory analysis of samples**

Media used to store samples was Remel MicroTestTM M4RT© (Thermo Fisher Scientific, Lenexa, KS) and samples were transported within 72 hours via cooler (maintained at 4° C) to the University of Pittsburgh Medical Center Clinical Virology Laboratory. The lab preserved 200ul of the M4 sample aliquots in 800ul lysis buffer (Biomerieux, Durham NC) and stored in the -80 °C freezer prior to sample extraction with the EasyMag (Biomerieux), a magnetic silicon-based extractor. Samples were processed as a batch.

The nucleic acid or eluates were spiked with Genmark Diagnostic’s (Carlsbad, CA) internal control MS2 prior to testing on the eSensor XT-8 instrument (Luminex, Austin, TX) using the respiratory viral panel or RVP-RUO panel. Tests were performed for both influenza A and B, and included subtyping of H1 and H3 influenza A viruses. Testing was also performed for respiratory syncytial virus (RSV A,B), parainfluenza viruses 1-4 (PIV 1-4), human metapneumovirus (HMPV), human rhinovirus (HRV), adenovirus (ADNO B,C,E), and coronavirus (CV 229E,NKL63, HKU1, OC43).

The overall sensitivity of the GenMark RVP platform has been estimated as 98.3% (95.5, 99.5) (Popowitch, Elena B., Stacey S. O'Neill, and Melissa B. Miller. "Comparison of the Biofire FilmArray RP, Genmark eSensor RVP, Luminex xTAG RVPv1, and Luminex xTAG RVP fast multiplex assays for detection of respiratory viruses." Journal of clinical microbiology 51.5 (2013): 1528-1533.) with virus specific sensitivities ranging from 100% (88.2, 100) (Adenovirus) to 90.7 (77.8, 96.9) (rhinovirus). The specificity of the assay across viruses was measured as 99.2% (95.4, 100) in the same reference with high specificities across all viruses.

**Table S0. Demographic characteristics of study schools. We present the number of participating and observed students. Distinction is made between kindergarten students enrolled in full-time and half-day programs in schools which operate such programs.**

| **District** | **School** | **Grade** | **Sex** | **Count** |
| --- | --- | --- | --- | --- |
| A | A1 | K | M | 14 |
|  |  | K | F | 13 |
|  |  | 1 | M | 11 |
|  |  | 1 | F | 15 |
|  |  | 2 | M | 20 |
|  |  | 2 | F | 12 |
|  |  | 3 | M | 11 |
|  |  | 3 | F | 10 |
|  |  | 4 | M | 10 |
|  |  | 4 | F | 22 |
|  |  | 5 | M | 13 |
|  |  | 5 | F | 17 |
|  |  | 6 | M | 18 |
|  |  | 6 | F | 18 |
| A | A2 | 9 | M | 27 |
|  |  | 9 | F | 34 |
|  |  | 10 | M | 23 |
|  |  | 10 | F | 27 |
|  |  | 11 | M | 25 |
|  |  | 11 | F | 20 |
|  |  | 12 | M | 2 |
|  |  | 12 | F | 3 |
| A | A3 | K | M | 18 |
|  |  | K | F | 9 |
|  |  | 1 | M | 11 |
|  |  | 1 | F | 16 |
|  |  | 2 | M | 14 |
|  |  | 2 | F | 16 |
|  |  | 3 | M | 20 |
|  |  | 3 | F | 15 |
|  |  | 4 | M | 24 |
|  |  | 4 | F | 10 |
|  |  | 5 | M | 19 |
|  |  | 5 | F | 13 |
|  |  | 6 | M | 16 |
|  |  | 6 | F | 17 |
|  |  | 7 | M | 20 |
|  |  | 7 | F | 14 |
|  |  | 8 | M | 13 |
|  |  | 8 | F | 17 |
| B | B1 | K | M | 0 |
|  |  | K | F | 1 |
|  |  | K (half) | M | 17 |
|  |  | K (half) | F | 22 |
|  |  | 1 | M | 14 |
|  |  | 1 | F | 26 |
|  |  | 2 | M | 17 |
|  |  | 2 | F | 19 |
|  |  | 3 | M | 29 |
|  |  | 3 | F | 21 |
|  |  | 4 | M | 19 |
|  |  | 4 | F | 16 |
| B | B2 | 5 | M | 62 |
|  |  | 5 | F | 61 |
|  |  | 6 | M | 85 |
|  |  | 6 | F | 62 |
| B | B3 | K | M | 7 |
|  |  | K | F | 2 |
|  |  | K (half) | M | 14 |
|  |  | K (half) | F | 24 |
|  |  | 1 | M | 23 |
|  |  | 1 | F | 20 |
|  |  | 2 | M | 26 |
|  |  | 2 | F | 28 |
|  |  | 3 | M | 18 |
|  |  | 3 | F | 23 |
|  |  | 4 | M | 31 |
|  |  | 4 | F | 24 |
| B | B4 | K | M | 2 |
|  |  | K | F | 4 |
|  |  | K (half) | M | 15 |
|  |  | K (half) | F | 15 |
|  |  | 1 | M | 21 |
|  |  | 1 | F | 13 |
|  |  | 2 | M | 20 |
|  |  | 2 | F | 24 |
|  |  | 3 | M | 17 |
|  |  | 3 | F | 16 |
|  |  | 4 | M | 23 |
|  |  | 4 | F | 18 |
| B | B5 | K | M | 6 |
|  |  | K | F | 6 |
|  |  | K (half) | M | 26 |
|  |  | K (half) | F | 29 |
|  |  | 1 | M | 38 |
|  |  | 1 | F | 22 |
|  |  | 2 | M | 31 |
|  |  | 2 | F | 28 |
|  |  | 3 | M | 45 |
|  |  | 3 | F | 40 |
|  |  | 4 | M | 43 |
|  |  | 4 | F | 28 |
| B | B6 | K | M | 0 |
|  |  | K | F | 0 |
|  |  | K (half) | M | 15 |
|  |  | K (half) | F | 20 |
|  |  | 1 | M | 24 |
|  |  | 1 | F | 13 |
|  |  | 2 | M | 23 |
|  |  | 2 | F | 22 |
|  |  | 3 | M | 24 |
|  |  | 3 | F | 12 |
|  |  | 4 | M | 18 |
|  |  | 4 | F | 18 |

**Table S1. Grade-specific contact rates.** Values are the mean averages reported by students in a cross-sectional contact diary study during 2011-12 from schools in the same school districts (A and B). Students were asked to complete contact diaries in which they listed all individuals with whom they had a face-to-face conversation or had skin-on-skin contact during a school day. The numbers used here are the subset of those encounters which occurred in school. For further information please see Grantz, Kyra H., et al. "Age-specific social mixing of school-aged children in a US setting using proximity detecting sensors and contact surveys." medRxiv (2020) <https://www.medrxiv.org/content/10.1101/2020.07.12.20151696v1>

.

| **Grade** | **Encounters** |
| --- | --- |
| K | 2.037 |
| 1 | 1.389 |
| 2 | 2.655 |
| 3 | 2.206 |
| 4 | 4.720 |
| 5 | 1.756 |
| 6 | 4.151 |
| 7 | 7.645 |
| 8 | 6.356 |
| 9 | 7.328 |
| 10 | 5.007 |
| 11 | 5.042 |
| 12 | 5.812 |

**Table S2.** Unobservable students with (enrolled students for whom we could not complete a telephone follow-up upon their absence) and number of absence and characterizable absence events made by enrolled students, stratified by school, grade and sex.

|  |  | **% Students eligible for free or reduced-price lunch** | **Enrolled students** | **Enrolled students with absences** | **Enrolled students with absences for whom follow-up was unachievable (% of those with absences)** | **Number of absence events** | **Number of characterizable absence events (%)** |
| --- | --- | --- | --- | --- | --- | --- | --- |
| **School** | *District A* |  |  |  |  |  |  |
|  | A1 | 72.1 | 295 | 226 | 91 (40.3) | 722 | 491 (68.0) |
|  | A2 | 72.1 | 257 | 208 | 96 (46.2) | 896 | 592 (66.1) |
|  | A3 | 86.5 | 375 | 236 | 93 (39.4) | 461 | 301 (65.3) |
|  | *District B* |  |  |  |  |  |  |
|  | B1 | 12.1 | 231 | 153 | 30 (19.6) | 328 | 280 (85.4) |
|  | B2 | 24.9 | 329 | 253 | 59 (23.3) | 646 | 533 (82.5) |
|  | B3 | 37.4 | 255 | 163 | 15 (9.2) | 291 | 270 (92.8) |
|  | B4 | 12.7 | 198 | 126 | 10 (7.9) | 278 | 258 (92.8) |
|  | B5 | 31.2 | 381 | 265 | 39 (14.7) | 729 | 669 (91.8) |
|  | B6 | 13.6 | 198 | 145 | 9 (6.2) | 369 | 356 (96.5) |
| **Grade** | K | - | 314 | 166 | 35 (21.1) | 398 | 336 (84.4) |
|  | 1 | - | 313 | 241 | 46 (19.1) | 628 | 541 (86.1) |
|  | 2 | - | 343 | 247 | 43 (17.4) | 556 | 489 (87.9) |
|  | 3 | - | 355 | 248 | 54 (21.8) | 606 | 498 (82.2) |
|  | 4 | - | 351 | 251 | 47 (18.7) | 575 | 494 (85.9) |
|  | 5 | - | 227 | 167 | 42 (25.1) | 466 | 355 (76.2) |
|  | 6 | - | 275 | 201 | 59 (29.4) | 517 | 401 (77.6) |
|  | 7 | - | 46 | 28 | 12 (42.9) | 51 | 27 (52.9) |
|  | 8 | - | 38 | 18 | 8 (44.4) | 27 | 17 (63.0) |
|  | 9 | - | 88 | 70 | 27 (38.6) | 306 | 232 (75.8) |
|  | 10 | - | 90 | 71 | 40 (56.3) | 279 | 153 (54.8) |
|  | 11 | - | 67 | 56 | 22 (39.3) | 255 | 178 (69.8) |
|  | 12 | - | 12 | 11 | 7 (63.6) | 56 | 29 (51.8) |
| **Sex** | Female | - | 1193 | 825 | 198 (24.0) | 2301 | 1847 (80.3) |
|  | Male | - | 1326 | 950 | 244 (25.7) | 2419 | 1903 (78.7) |

**Model selection**

We used a forward selection process to identify covariates that best explained our observed pattern of infection across the participants. The Selection process began from an initial model which only included an intercept term. Candidate variables were systematically considered, and model complexity increased only if deemed significant through a likelihood ratio test.

Candidate variables included: length of school instruction (halfday); school grade as a linear term (grade); sex; vaccination status (vacc); grade-specific social contact rate (Kschool) (Table S1); percentage of students on free or reduced price lunch (lunch) (Table S2); class size (class.size); 1 denotes an intercept term. Social contact mixing rate was collected as part of a parallel study, and is a measure of the average daily number of different people encountered by students in grade with the school setting (see Table S1). This variable was included as a candidate variable as contact rate may be associated with risk of infection. Mixed effect logistic regression models were used throughout, with a nested random effect term of school nested within district. p-values refer to the significance of a likelihood ratio test between that model and the model from the previous step. At each iteration, the model with lowest AIC was selected and progression to the next step was conditional on a likelihood ratio test p-value<0.05. The selection process for each outcome variable is shown in Table S3.

The observed pattern of ILI cases was best explained by a model that contained terms for duration of attendance, self-reported vaccination status, and grade. When the outcome was testing positive for any of the candidate respiratory viruses or testing positive for either influenza type, the selected model contained terms for duration of attendance and grade. For testing positive for influenza A, the selected model only included terms for the number of people encountered within school (a protective association, OR 0.78, 95% CI 0.63-0.97). For testing positive for influenza B, the selected model contained a term for duration of attendance, though we note a model also including a term for grade narrowly missed being selected (Table S4).

**Table S3**. Forward model selection process. Final selected models are highlighted in bold. Variable names: halfday = whether students have truncated attendance; lunch = the percentage of students in the school eligible for free or reduced lunches; vacc = self-reported vaccination status; grade = ordinal school grade; sex = student sex; K_school = grade-specific contact rate.

| **Outcome variable** | **Step** | **Model** | **AIC** | **ΔAIC** | **p-value** |
| --- | --- | --- | --- | --- | --- |
| Influenza-like illness | 1 | 1 | 2042.172004 | NA | NA |
|  | 2 | 1 + halfday | 2029.197637 | -12.97436755 | 0.000108982 |
|  | 3 | 1 + halfday + lunch | 2022.530525 | -6.667111964 | 0.003240043 |
|  | 4 | 1 + halfday + lunch + vacc | 2016.865608 | -5.664917277 | 0.007966909 |
|  | **5** | **1 + halfday + lunch + vacc + grade** | **2014.502533** | **-2.363075087** | **0.036725954** |
|  | 6 | 1 + halfday + lunch + vacc + grade + sex | 2015.764827 | 1.262294748 | 0.390396961 |
| Any respiratory virus^%^ | 1 | 1 | 1596.555027 | NA | NA |
|  | 2 | 1 + halfday | 1582.74103 | -13.81399655 | 6.99E-05 |
|  | 3 | 1 + halfday + lunch | 1575.341691 | -7.399338568 | 0.002170637 |
|  | **4** | **1 + halfday + lunch + grade** | **1570.663431** | **-4.678260022** | **0.009759585** |
|  | 5 | 1 + halfday + lunch + grade + vacc | 1570.125806 | -0.537625492 | 0.103434911 |
| influenza A or B | 1 | 1 | 1195.390427 | NA | NA |
|  | 2 | 1 + halfday | 1182.681154 | -12.70927319 | 0.000125428 |
|  | 3 | 1 + halfday + grade | 1175.316224 | -7.36493016 | 0.002211764 |
|  | **4** | **1 + halfday + grade + lunch** | **1168.710249** | **-6.605974805** | **0.00335062** |
|  | 5 | 1 + halfday + grade + lunch + K_school | 1168.982529 | 0.272279894 | 0.188702323 |
| influenza A | 1 | 1 | 519.8688733 | NA | NA |
|  | **2** | **1 + K_school** | **515.9552304** | **-3.913642933** | **0.015024072** |
|  | 3 | 1 + K_school + halfday | 516.5820643 | 0.626833887 | 0.241268646 |
| influenza B | 1 | 1 | 942.3885904 | NA | NA |
|  | 2 | 1 + halfday | 929.5642908 | -12.82429956 | 0.000118005 |
|  | 3 | 1 + halfday + lunch | 924.048273 | -5.516017792 | 0.006115272 |
|  | **4** | **1 + halfday + lunch + grade** | **921.8329858** | **-2.215287241** | **0.040061283** |
|  | 5 | 1 + halfday + lunch + grade + K_school | 920.2669701 | -1.566015733 | 0.05897365 |

^%^ Influenza A/H1, influenza A/H3, influenza B, RSV A, B, picornavirus 1, 2, 3, 4, metapneumovirus, rhinovirus detected, adenovirus B, C, E, coronavirus 229E, NL63, HKU1, OC43 detected using Genmark Diagnostic’s RVP-RUO panel.

**Table S4**. Final models identified using the forward selection process. Hierarchical random intercepts were included for district, and school (within district. Candidate variables included: length of school instruction; grade (linear term); sex; vaccination status; grade-specific social contact rate. Variables significant at the 95% level are shown in bold. Contact rates are the average (mean) number of contacts by grade reported by students. Total number of observations = 2077.

|  |  | **Response variable^*^** | | | | | | | |
| --- | --- | --- | --- | --- | --- | --- | --- | --- | --- |
|  |  | **Symptomatic infection** | **Laboratory confirmed infection** | | | | | | |
|  |  | **ILI** | **Any respiratory virus^%^** | | **Influenza (A or B)** | | **Influenza A** | **Influenza B** | |
| **Random effect variables** | | Percent of variance attributable to random effect^ | | | | | | | |
| school  district  residuals | | 0.00  43.38  56.62 | 0.00  33.28  66.72 | | 0.00  34.33  65.67 | | 8.37  0.00  91.63 | 11.11  0.00  88.89 | |
| **Fixed effect variables** | | Odds ratio (95% Confidence Interval) | | | | | | | |
| Intercept | | 2.65 (0.63-11.08) | | 1.22 (0.32-4.60) | | 1.12 (0.25-4.95) | 0.06 (0.03-0.11) | | 0.25 (0.13-0.47) |
| % students in school eligible for free or reduced lunch | | 0.96 (0.95-0.97) | | 0.97 (0.95-0.98) | | 0.96 (0.94-0.98) | - | | 0.98 (0.96-0.99) |
| Duration of attendance  Full day (n=1880)  Half day (n=197) | | 1  0.35 (0.22-0.56) | | 1  0.28 (0.15-0.50) | | 1  0.21 (0.10-0.44) | -  - | | 1  0.19 (0.08-0.45) |
| Grade (linear term) | | 0.95 (0.90-1.00) | | 0.91 (0.85-0.97) | | 0.85 (0.78-0.92) | - | | 0.88 (0.78-0.99) |
| Sex  Male (n=1082)  Female (n=995) | | -  - | | -  - | | -  - | -  - | | -  - |
| Vaccination  No (n=850)  Yes (n=694)  Not reported (533) | | 1  0.68 (0.53-0.89)  0.76 (0.58-1.00) | | -  -  - | | -  -  - | -  -  - | | -  -  - |
| Grade-specific contact rate  (linear term) | | - | | - | | - | 0.78 (0.63-0.96) | | - |

$ Odd ratios were calculated using random effect logistic regression

^%^ Influenza A/H1, influenza A/H3, influenza B, RSV A, B, picornavirus 1, 2, 3, 4, metapneumovirus, rhinovirus detected, adenovirus B, C, E, coronavirus 229E, NL63, HKU1, OC43 detected using Genmark Diagnostic’s RVP-RUO panel.

^ Percentage of variance attributable to each random effect was calculated by dividing the standard deviation of each component by the total

^%^ Influenza A/H1, influenza A/H3, influenza B, RSV A, B, picornavirus 1, 2, 3, 4, metapneumovirus, rhinovirus detected, adenovirus B, C, E, coronavirus 229E, NL63, HKU1, OC43 detected using Genmark Diagnostic’s RVP-RUO panel.

^ Percentage of variance attributable to each random effect was calculated by dividing the standard deviation of each component by the total

**Table S5**. Odds ratios^$^ of ILI, any virus, influenza (A or B), influenza A, influenza B on grade within all schools where kindergarteners were taught for half-days (schools B1, B3, B4, B5, and B6).

|  |  | **Response variable** | | | | |
| --- | --- | --- | --- | --- | --- | --- |
|  |  | **Symptomatic infection** | **Laboratory confirmed infection** | | | |
|  |  | **ILI** | **Any respiratory virus^%^** | **Influenza (A or B)** | **Influenza A** | **Influenza B** |
| **Random effect variables** | | Percent of variance attributable to random effect^ | | | | |
| School  Residuals | | 9.4  90.6 | 10.3  89.7 | 12.0  88.0 | 9.3  90.7 | 16.2  83.8 |
| **Fixed effect variables** | | Odds ratio (95% Confidence Interval) | | | | |
| Intercept | | **0.14 (0.09-0.23)** | **0.08 (0.05-0.15)** | **0.05 (0.03-0.10)** | **0.02 (0.01-0.06)** | **0.03 (0.01-0.07)** |
| Grade  K  1  2  3  4 | | 1  **2.61 (1.58-4.31)**  **2.59 (1.58-4.23)**  **1.86 (1.12-3.08)**  **2.17 (1.32-3.58)** | 1  **3.44 (1.90-6.23)**  **2.93 (1.62-5.29)**  **2.19 (1.19-4.02)**  **2.31 (1.26-4.25)** | 1  **3.67 (1.80-7.45)**  **3.26 (1.60-6.61)**  **2.51 (1.22-5.17)**  **3.01 (1.47-6.15)** | 1  2.61 (0.91-7.52)  2.16 (0.74-6.31)  0.36 (0.07-1.85)  0.76 (0.20-2.86) | 1  **3.91 (1.65-9.27)**  **3.99 (1.70-9.36)**  **3.70 (1.57-8.69)**  **4.14 (1.77-9.70)** |

$ Odd ratios were calculated using mixed effect logistic regression using a random effect for school, fixed variables are grade.

^%^ Influenza A/H1, influenza A/H3, influenza B, RSV A, B, picornavirus 1, 2, 3, 4, metapneumovirus, rhinovirus detected, adenovirus B, C, E, coronavirus 229E, NL63, HKU1, OC43 detected using Genmark Diagnostic’s RVP-RUO panel.

**Table S6**. Odds ratios^$^ of ILI, any virus, influenza (A or B), influenza A, influenza B on duration of instruction within schools with any kindergarteners taught for half-days (schools B1, B3, B4, B5, and B6).

|  |  | **Response variable** | | | | |
| --- | --- | --- | --- | --- | --- | --- |
|  |  | **Symptomatic infection** | **Laboratory confirmed infection** | | | |
|  |  | **ILI** | **Any respiratory virus^%^** | **Influenza (A or B)** | **Influenza A** | **Influenza B** |
| **Random effect variables** | | Percent of variance attributable to random effect^ | | | | |
| School  Residuals | | 20.8  79.3 | 35.6  64.4 | 82.1  17.9 | 31.3  68.7 | 68.1  31.9 |
| **Fixed effect variables** | | Odds ratio (95% Confidence Interval) | | | | |
| Intercept | | **0.20 (0.06-0.65)** | **0.16 (0.04-0.65)** | 0.11 (0.01-1.14) | **0.04 (0.00-0.34)** | **0.05 (0.00-0.54)** |
| Duration of attendance  Full day  Half day | | 1  0.66 (0.20-2.22) | 1  0.40 (0.09-1.73) | 1  0.16 (0.02-1.48) | 1  0.44 (0.03-5.46) | 1  0.35 (0.03-4.95) |

$ Odd ratios were calculated using mixed effect logistic regression using a random effect for school, fixed variables are grade.

^%^ Influenza A/H1, influenza A/H3, influenza B, RSV A, B, picornavirus 1, 2, 3, 4, metapneumovirus, rhinovirus detected, adenovirus B, C, E, coronavirus 229E, NL63, HKU1, OC43 detected using Genmark Diagnostic’s RVP-RUO panel.

**Table S7**. Odds ratios^$^ of ILI, any virus, influenza (A or B), influenza A, influenza B on length of school instruction, grade, sex, and self-reported vaccination status among schoolchildren in schools with both half and full day Kindergarteners (schools B1, B3, B4, and B5). As all the schools belonged to the same school district, only a random effect term for school was included in the models. Variables significant at the 95% level are shown in bold.

|  |  | **Response variable** | | | | |
| --- | --- | --- | --- | --- | --- | --- |
|  |  | **Symptomatic infection** | **Laboratory confirmed infection** | | | |
|  |  | **ILI** | **Any respiratory virus^%^** | **Influenza (A or B)** | **Influenza A** | **Influenza B** |
| **Random effect variables** | | Percent of variance attributable to random effect^ | | | | |
| School  Residuals | | 8.6  91.4 | 10.1  89.9 | 13.4  86.6 | 9.8  90.2 | 15.0  85.0 |
| **Fixed effect variables** | | Odds ratio (95% Confidence Interval) | | | | |
| Intercept | | **0.52 (0.30-0.89)** | **0.34 (0.19-0.62)** | **0.22 (0.11-0.44)** | **0.16 (0.06-0.40)** | **0.12 (0.05-0.25)** |
| Duration of attendance  Full day  Half day | | 1  **0.25 (0.13-0.48)** | 1  **0.15 (0.06-0.37)** | 1  **0.09 (0.03-0.31)** | 1  **0.10 (0.02-0.48)** | 1  **0.11 (0.02-0.50)** |
| Grade (linear term) | | 0.90 (0.78-1.04) | 0.87 (0.74-1.02) | 0.90 (0.75-1.07) | **0.59 (0.43-0.83)** | 1.01 (0.83-1.24) |
| Sex  Male  Female | | 1  0.85 (0.62-1.17) | 1  0.91 (0.63-1.31) | 1  1.01 (0.67-1.52) | 1  0.99 (0.48-2.04) | 1  0.95 (0.60-1.50) |
| Vaccination  No  Yes  Not reported | | 1  **0.66 (0.46-0.96)**  0.71 (0.47-1.08) | 1  0.80 (0.53-1.21)  0.75 (0.46-1.22) | 1  0.77 (0.48-1.22)  0.75 (0.44-1.30) | 1  0.56 (0.24-1.28)  0.47 (0.17-1.28) | 1  0.86 (0.51-1.45)  0.87 (0.48-1.60) |

$ Odd ratios were calculated using mixed effect logistic regression using a random effect for school, fixed variables are grade.

^%^ Influenza A/H1, influenza A/H3, influenza B, RSV A, B, picornavirus 1, 2, 3, 4, metapneumovirus, rhinovirus, adenovirus B, C, E, coronavirus 229E, NL63, HKU1, OC43 detected using Genmark Diagnostic’s RVP-RUO panel.

**Table S8**. Association between testing positive according to four different outcomes and delay in swabbing (delay between symptom onset and swab taken). Odds ratios and p-values from logistic regression models are shown. There is no evidence that delay in swabbing reduces the probability of a test returning a negative result for the time scales measured in the study.

|  |  | | **Odds ratio** | | |  |
| --- | --- | --- | --- | --- | --- | --- |
|  |  | | *Estimate* | *95% CI lower* | *95% CI upper* | p-value |
| **Infection outcome** | | *Any virus* | 0.986 | 0.919 | 1.059 | 0.706 |
|  |  | *Influenza* | 1.000 | 0.932 | 1.072 | 0.998 |
|  |  | *Influenza A* | 0.890 | 0.789 | 1.004 | 0.058 |
|  |  | *Influenza B* | 1.051 | 0.976 | 1.132 | 0.186 |

**Figure S1**. Cumulative attack rate for influenza type A and B, by grade and district. Lines denote binomial 95% confidence intervals.

**Figure S2.** Cumulative attack rates, stratified by grade and school, for other respiratory viruses. Lines denote binomial 95% confidence intervals.

**Figure S3.** Cumulative attack rate (CAR) by infection outcome (columns), stratified by grade and school (rows).


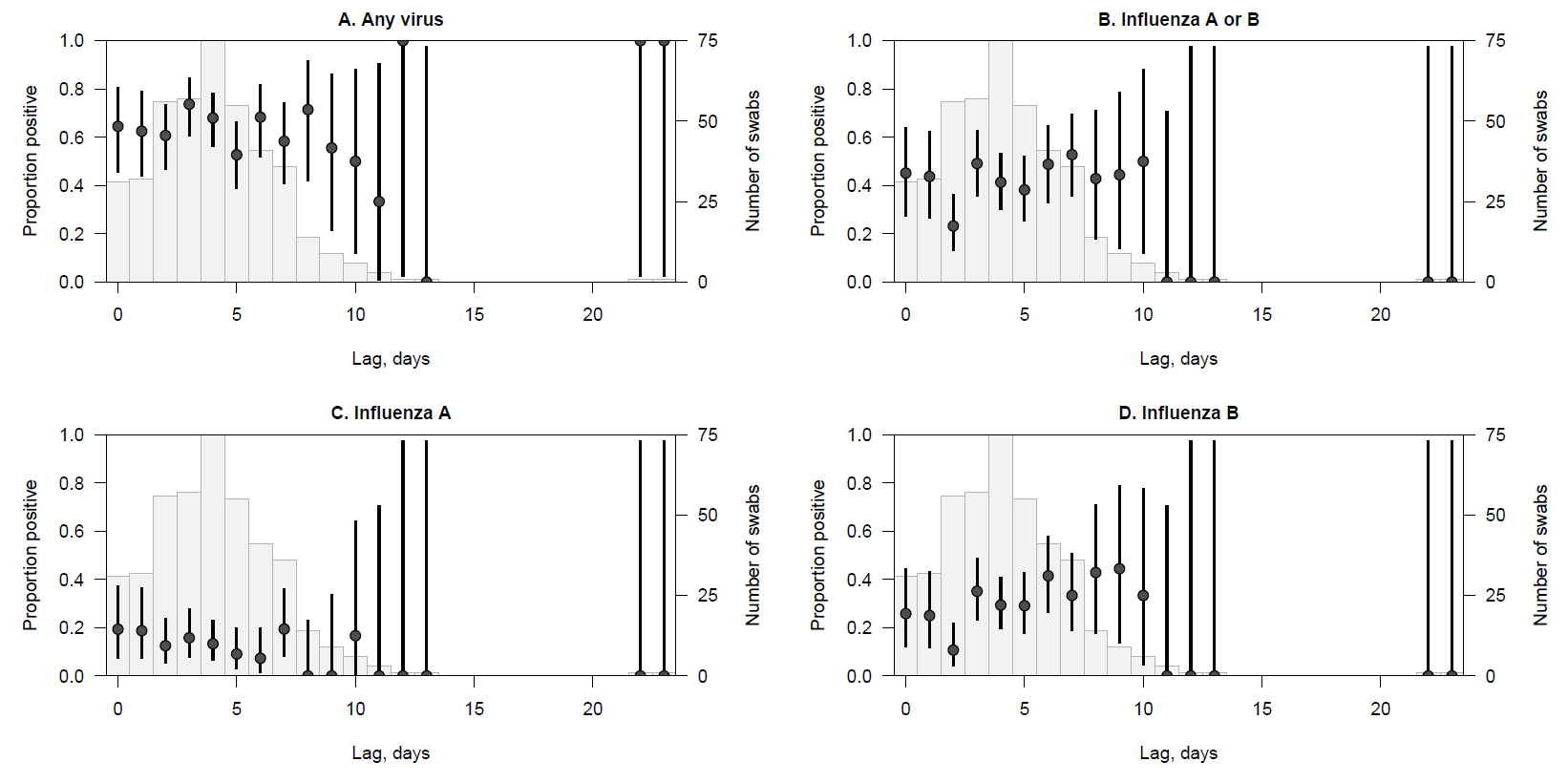


**Figure S4.** Relationship between the proportion of samples testing positive for virus and the delay between symptom onset date and swabbing date. Circles denote proportions; lines denote 95% binomial confidence intervals. Pale grey bars indicate the number of swabs taken for a given lag.


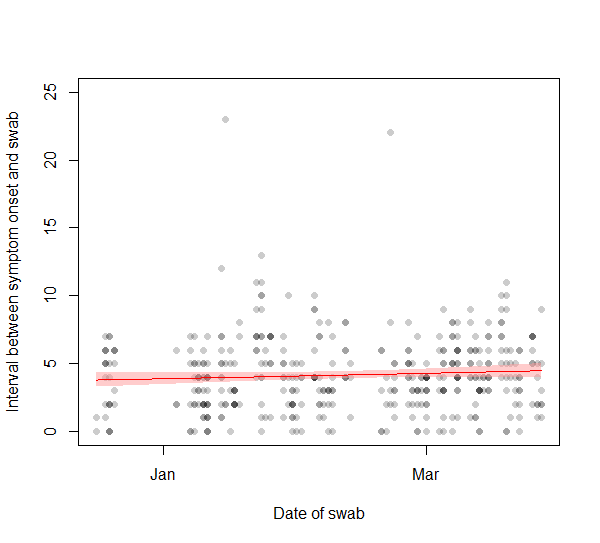


**Figure S5** Spline of secular time fitted using a negative binomial GAM of swabbing delay. There was no statistical support for a nonlinear relationship between the interval and secular time (the effective degrees of freedom of the selected spline was 1); the linear term is not significant, indicating no trend in swabbing delay during the course of the study.
